# Chronic kidney disease: prevalence and association with handgrip strength in a cross-sectional study

**DOI:** 10.1186/s12882-021-02452-5

**Published:** 2021-07-02

**Authors:** Yang Cheng, Min Liu, Yu Liu, Haifeng Xu, Xiaotian Chen, Hui Zheng, Xiaojun Wu, Zhixiang Shen, Chong Shen

**Affiliations:** 1 Center for Health Management, Geriatric Hospital of Nanjing Medical University, 65 Jiangsu Road, 21009 Nanjing, China; 2 Department of Chronic Non-communicable Diseases Control, Center for Disease Control and Prevention of Jurong City, 212400 Jurong, China; 3 Department of Clinical Epidemiology, Children’s Hospital of Fudan University, 201102 Shanghai, China; 4 People’s Hospital of Jurong City, 212400 Jurong, China; 5 Department of Epidemiology, School of Public Health, Nanjing Medical University, 101 Longmian Avenue, 211166 Nanjing, China; 6 Division of Clinical Epidemiology, Geriatric Hospital of Nanjing Medical University, 210009 Nanjing, China

**Keywords:** Handgrip strength, chronic kidney disease, Chinese community-dwelling persons

## Abstract

**Background:**

Poor physical function is strongly associated with mortality and poor clinical outcomes in adults with chronic kidney disease (CKD). Handgrip strength (HGS) is an important index for physical function in the general population, and the association between HGS and CKD is worth investigating.

**Methods:**

From September to November 2015, we conducted a cross-sectional study consisting of 10,407 participants in Jurong City, China. Age-related and sex-specific HGS percentile curves were constructed using the GAMLSS method. In addition, logistic regression was applied to estimate the association between HGS and the presence of CKD with odds ratios (ORs) and 95 % confidence intervals (CIs).

**Results:**

Participants with low HGS tended to be older and were more likely to have CKD (8.73 %). Smoothed centile curves of HGS showed a similar shape in both sexes: participants peaked at approximately 20–35 years old and gradually decreased after the age of 50. In addition, independent of age and other factors, the decreased presence of CKD was significantly identified in individuals with moderate (OR: 0.64, 95 % CI: 0.49–0.83) and high HGS (OR: 0.37, 95 % CI: 0.23–0.58).

**Conclusions:**

We concluded that HGS was significantly negatively associated with CKD in Chinese community-dwelling persons.

**Supplementary Information:**

The online version contains supplementary material available at 10.1186/s12882-021-02452-5.

## Background

In 2012, the chronic kidney disease (CKD) prevalence in the general adult population was approximately 10.8 % in mainland China [1]. Once renal failure occurs, dialysis and transplantation are the only available treatments [2–5] and lead to high medical costs and a heavy disease burden [6]. Thus, there is an urgent need to identify the early indicators of CKD.

Patients with CKD typically report lower measures of physical performance than the general population [7–10]. A sedentary lifestyle commonly commences in the early stages of CKD, which, in turn, leads to a decrease in physical performance, accompanied by a decline in glomerular filtration rate [11, 12]. In addition, CKD  has systemic effects such as loss of appetite, chronic inflammation, anemia, metabolic acidosis and so on, all of which contribute to a loss of muscle mass and decline in physical performance [8, 11, 12].

Handgrip strength (HGS) is an easily obtainable metric and noninvasive method for evaluating extremity strength and function and is commonly used to reflect overall muscle strength in the general population [13, 14]. However, the relationship between renal function and HGS has not been fully investigated.

In this study, we aimed to evaluate the association between HGS and the presence of CKD in a relatively large sample from a Chinese population. The results will help to better understand muscle function and physical performance in the prediction of CKD.

## Methods

### Study participants

Jurong is a country-level city under the jurisdiction of Zhenjiang city, located in the south of Jiangsu Province. The present cross-sectional study involved multistage sampling among people aged ≥ 18 years, and the participants were surveyed from September to November 2015. A total of 11,151 adults comprising 4,388 men and 6,762 women were enrolled in this study. We excluded samples with missing information on sex (n = 1), body mass index (BMI, n = 2), smoking status (n = 1), drinking status (n = 2), HGS (n = 427), diastolic pressure (n = 2), blood glucose (n = 73), estimated glomerular filtration rate (eGFR, n = 1) and those reporting a prior history of other kidney diseases (n = 235). Thus, 10,407 individuals (men = 4,084; women = 6,323) were included in the final data analysis. The Institutional Review Board of the Geriatric Hospital of Nanjing Medical University approved this study, and written informed consent was obtained at recruitment. All methods for the population study were carried out in accordance with the relevant guidelines and regulations of the Geriatric Hospital of Nanjing Medical University.

### Anthropometric and physiological measurements

According to standard procedures, baseline measurements of height, weight, blood pressure (BP), and HGS were obtained by trained staff. BMI was calculated as weight (kg)/square of height (m^2^). HGS was measured in kilograms using a CAMRY electronic hand dynamometer (EH101, CAMRY, Zhongshan, China) in a standing position with the arm extended straight down to the side. The participant was told to apply the maximum grip strength twice with both the left and right hands. A resting interval of at least 30 s was allowed between each measurement. The maximal measured HGS was used in the analysis. In our study, the measurements for the average HGS of the right and the left hand were used to assess muscle strength. The measured HGS was further divided into sex-specific tertiles of HGS (to obtain equal distributions of men and women) as follows: tertile 1: ≤31.00 (men) and ≤ 21.05 (women) kg; tertile 2: 31.00-38.85 (men) and 21.05–25.70 (women) kg; tertile 3: ≥38.85 (men) and ≥ 25.70 (women) kg.

### Biochemistry measurements

After 8 h of overnight fasting, five milliliters of venous blood was drawn by a research nurse to measure serum glucose (GLU); the lipid profile: high-density lipoprotein cholesterol (HDL-C), low-density lipoprotein cholesterol (LDL-C), serum triglyceride (TG) and total cholesterol (TC); serum cystatin C (Scc) and serum creatinine (Scr). The eGFR was calculated based on Scr, Scc, age, and sex with the Chronic Kidney Disease Epidemiology Collaboration (CKD-EPI_2012_) Eqs. [15, 16]. Kidney function was classified as proposed by the National Kidney Foundation and merged into the following three categories: normal (eGFR: ≥ 90 mL/min/1.73 m^2^), mildly reduced (60 - <90 mL/min/1.73 m^2^), and moderately to severely reduced (< 60 mL/min/1.73 m^2^) [17]. In this study, we defined prevalent CKD patients as those with eGFR < 60mL/min/1.73 m^2^.

### Definition of hypertension and diabetes

Hypertension was defined as having systolic blood pressure ≥ 140 mmHg and/or diastolic blood pressure ≥ 90 mmHg or a self-reported diagnosis of hypertension. Type 2 diabetes mellitus was defined as GLU ≥ 7.0 mmol/L or a self-reported diagnosis of diabetes and the exclusion of type 1 diabetes [18].

### Data collection of other characteristics

A standard questionnaire was used to collect detailed information, including demographic characteristics (age and sex), medical history (hypertension and diabetes), personal behavior (smoking and drinking status), and Physical Activity Index (PAI) values [19], which were described in a previous study [20]. All the participants were interviewed face-to-face by trained staff, and once the interview was completed, the questionnaires were double-checked to ensure the accuracy of the information.

### Statistical analysis

Variables were tested for normality using the Lilliefors test, and then characteristics of the overall population were reported as the mean ± SD (normal distribution) or median with interquartile range (nonnormal distribution) or as a percentage, as appropriate. Comparisons between tertiles for continuous variables were conducted using one-way analysis of variance (ANOVA) (normal distribution) and the Kruskal–Wallis test (non-normal distribution). The chi-squared test was used to examine differences between categorical variables. P for trend was calculated through the Cochran-Armitage test or linear regression analyses. We implemented the General Additive Model for Location Scale and Shape (GAMLSS) method to calculate percentile curves of HGS by age through the gamlss package (version 5.1-5) [21]. The association analyses between each HGS index and the presence of CKD were identified by multiple logistic regression to estimate the odds ratio (OR).

To avoid multicollinearity among similar parameters (for example, BMI, HDL-C, LDL-C, TC and TG), we used the correlation matrix and variance inflation factor (VIF) statistics to identify the parameters with the best potential performance. A VIF > 10 was regarded to be indicative of autocorrelation.

Data analyses were performed with R software (Version 3.6.1; The R Foundation for Statistical Computing, http://www.cran.r-project.org/). A two-tailed *P*-value < 0.05 was considered statistically significant.

## Results

As shown in Table 1, 39.24 % of the participants were men with an age ranging from 18 to 90 years old, and the prevalence of CKD was 3.96 %. We found that individuals with higher HGS were more likely to be younger and physically active, to be current regular smokers and drinkers and to have higher BMI, TG and eGFR values than those with lower HGS (all *P* for trend < 0.001). We also found that individuals with higher HGS tended to be less likely to have CKD (low: 8.73 %, moderate: 2.50 %, and high: 0.69 %, *P* < 0.001).

**Table 1:** General characteristics of the subjects included in the study

Variables ^c^	All subjects(*n* = 10,407)	Sex-specific tertiles of handgrip strength			*P value*^*a*^	*P* for trend^b^
		**Low (*n* = 3,448)**	**Moderate (*n* = 3,481)**	**High (*n* = 3,478)**		
**Age range (year)**	18–95	20–95	18–87	20–85	< 0.001	< 0.001
**Gender man, n (%)**	4,084(39.24)	1,351(39.18)	1,365(39.21)	1,368(39.33)	0.991	0.898
**Women, n (%)**	6,323(60.76)	2,097(60.82)	2,116(60.79)	2,110(60.67)		
**BMI (kg/m**^**2**^**)**	24.75(22.55–27.06)	24.26(21.93–26.81)	24.64(22.48–26.94)	25.31 (23.20-27.45)	< 0.001	< 0.001
**PAI**	31.55(27.92–37.92)	30.61(26.95–35.90)	32.07(28.36–38.96)	32.68(28.45–39.29)	< 0.001	< 0.001
**Current regular smoker, n (%)**	2,311(22.21)	689(19.98)	778(22.35)	844(24.27)	< 0.001	< 0.001
**Current regular drinker, n (%)**	2,880(27.67)	808(23.43)	966(27.75)	1,106(31.80)	< 0.001	< 0.001
**Hypertension, n (%)**	6,185(59.43)	2,348(68.10)	2,027(58.23)	1,810(52.04)	< 0.001	< 0.001
**Diabetes, n (%)**	1,795(17.25)	711(20.62)	576(16.55)	508(14.61)	< 0.001	< 0.001
**Antihypertensive medication, n(%)**	3453(33.18)	1378(39.97)	1135(32.61)	940(27.03)	< 0.001	< 0.001
**Antidiabetic medication, n(%)**	863(8.29)	351(10.18)	308(8.85)	204(5.87)	< 0.001	< 0.001
**HDL-C(mmol/L)**	1.48(1.23–1.76)	1.51(1.25–1.79)	1.49(1.25–1.78)	1.43(1.18–1.70)	< 0.001	< 0.001
**LDL-C(mmol/L)**	2.82(2.34–3.35)	2.80(2.29–3.33)	2.85(2.37–3.36)	2.81(2.34–3.35)	0.019	0.041
**TG (mmol/L)**	1.30(0.94–1.79)	1.27(0.94–1.79)	1.30(0.93–1.85)	1.35(0.96–1.99)	< 0.001	< 0.001
**TC (mmol/L)**	5.03(4.46–5.65)	5.02(4.46–5.09)	5.06(4.49–5.67)	5.02(4.44–5.65)	0.053	0.001
**eGFR(ml/min/1.73m**^**2**^**)**	94.62(83.33-105.94)	87.62(77.10-99.39)	94.76(84.24-105.13)	101.03(89.89-111.05)	< 0.001	< 0.001
**CKD, n (%)**	412(3.96)	301(8.73)	87(2.50)	24(0.69)	< 0.001	< 0.001

^a^ Comparisons between groups analyzed by ANOVA or Kruskal–Wallis test for continuous variables ; and Chi-squared test was used to examine the differences for categorical variables;

^b^ P for trend was calculated through Cochran-Armitage test or linear regression analyses

^c^*BMI *Body mass index; *PAI* Physical Activity Index; *HDL-C* high density lipoprotein cholesterol; *LDL-C* low-density lipoprotein cholesterol; *TG* serum triglyceride; *TC* total cholesterol; *eGFR* estimated glomerular filtration rate; *CKD* chronic kidney disease

**Fig. 1:**
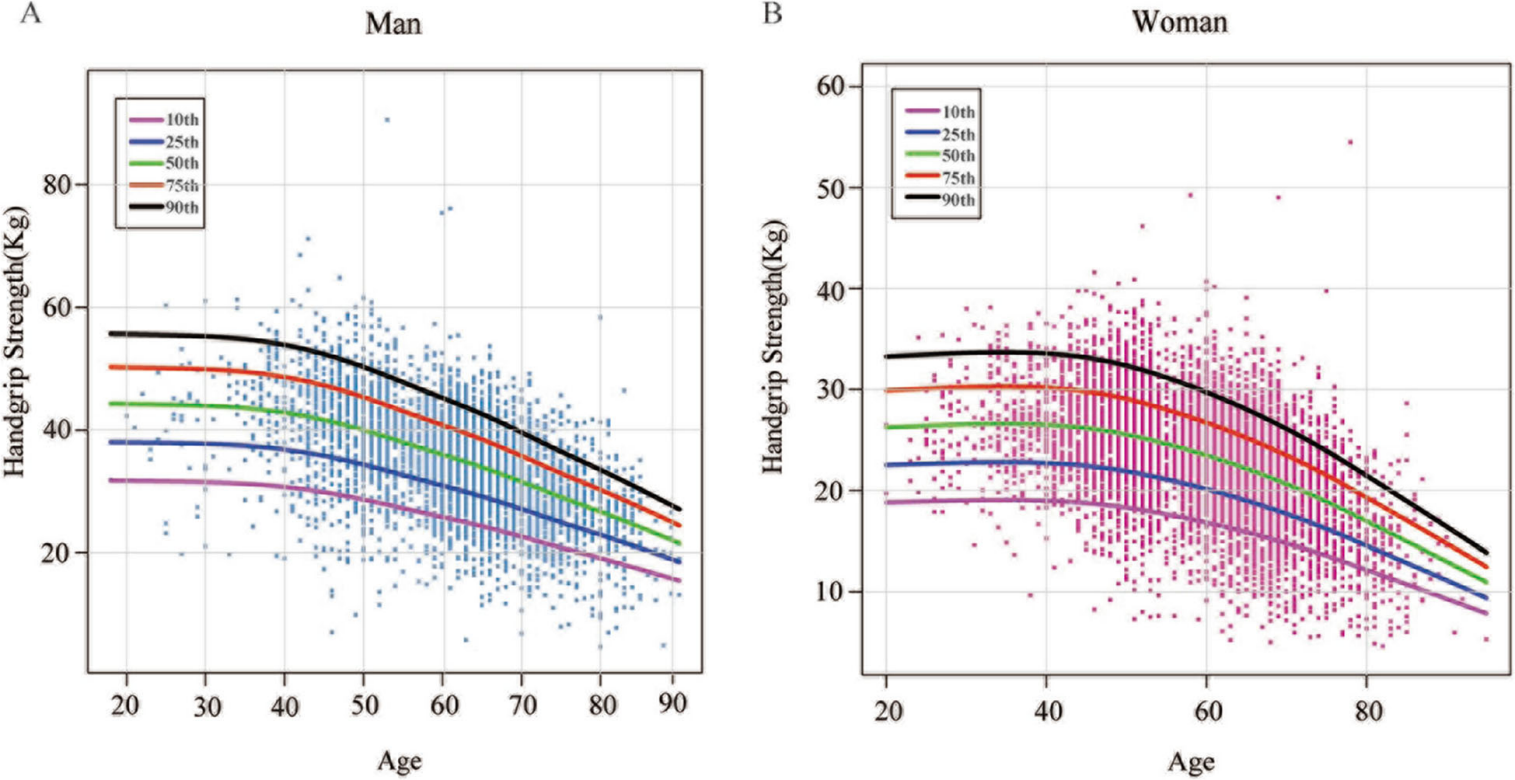
Age-related distribution of handgrip strength stratified by sex. Lines in different colors indicate the percentile as denoted (10-90th percentiles)

**Fig. 2:**
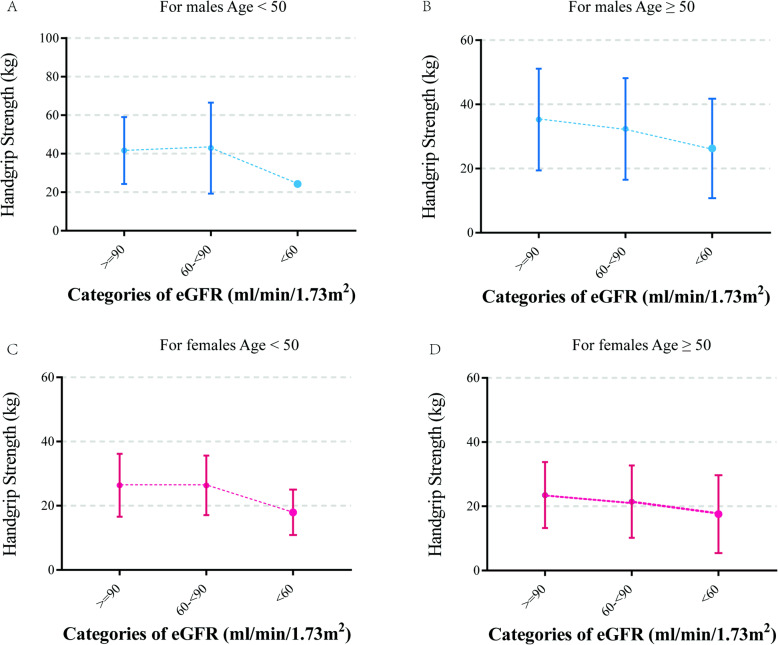
The mean values of handgrip strength according to categories of eGFR. Error bars represent 95 % confidence intervals. (**A**) For men under the age of 50; (**B**) for men aged 50 and above; (**C**) for women under the age of 50; (**D**) for women aged 50 and above

Figure 1 depicts the centile curves of HGS by age for males and females. We identified that the two sets of HGS percentiles were similar in shape: they increased gradually to peak in early adult life, stabilized in midlife and declined from midlife onwards, but the HGS levels were different. The 10th, 25th, 50th, 75th and 90th centiles of HGS for the two groups are also presented in Table S1. Generally, males were stronger on average than females; males’ median strength was 1.68 times that of females. Excitingly, we also identified that HGS in both sexes gradually decreased with age after the age of 50, and the decline was pronounced when the patient reached 60 years old. We further studied the distribution of HGS across categories of eGFR, as shown in Fig. 2. In both sexes before the age of 50, the average HGS was maintained at a relatively stable level in participants with eGFR ≥ 60 ml/min/1.73 m^2^, while in participants aged 50 and over, the average HGS showed a stepwise decrease with declining categories of eGFR levels. As expected, participants with lower eGFR were more likely to have lower HGS (Fig SA1-3).

Only one parameter was excluded because of collinearity. After adjusting for the remaining potential confounding factors (age, sex, smoking status, drinking status, PAI, BMI, HDL-C, LDL-C, TG, and history of hypertension and diabetes), moderate and high HGS were significantly associated with a 36 and 63 % decreased prevalence of CKD (moderate: 0.64 [0.49–0.83]; high: 0.37 [0.23–0.58] Table 2). In addition, the odds ratios for CKD were 1.00, 0.61 (0.41–0.90) and 0.31 (0.14–0.60) in men and 1.00, 0.67 (0.46–0.96) and 0.44 (0.23–0.77) in women after adjustment for confounders. We then performed stratification analysis of HGS by age, gender, smoking status, drinking status, history of hypertension and diabetes (Table 2). A stronger association was observed in the old participants aged 50 years old and above, obesity, smokers, drinkers, and subjects with high physical acitivity, history of hypertension and diabetes. To further evaluate the association between low HGS and the prevalence of CKD, we also set high HGS as the reference. We identified that the low HGS group showed higher ORs for CKD than did the moderate and high groups (low: OR 2.68, 95 % CI 1.74–4.30; moderate: OR 1.71, 95 % CI 1.08–2.79) (Table S2).

**Table 2:** Odds ratio and 95 % CI for CKD according to tertiles of handgrip strength

Characteristics	Sample	Handgrip strength		
	**(Case/Control)**	**Low (*n* = 3440)**	**Moderate(*n* = 3484)**^b^	**High (*n* = 3483)**^b^
**Entire**^a^	412/9,995	Ref	0.64(0.49–0.83)	0.37(0.23–0.58)
Gender				
Men	189/3,895	Ref	0.61(0.41–0.90)	0.31(0.14–0.60)
Women	223/6,100	Ref	0.67(0.46–0.96)	0.44(0.23–0.77)
**Age (≥ 50)**		Ref	0.35(0.27–0.44)	0.13(0.08–0.19)
Men	188/3,231	Ref	0.34(0.23–0.49)	0.12(0.06–0.21)
Women	221/4,750	Ref	0.35 (0.25–0.50)	0.14(0.08–0.24)
**BMI (≥ 28 kg/m**^**2**^**)**				
Yes	86/1,760	Ref	0.58(0.33–1.01)	0.29(0.12–0.66)
No	326/8,235	Ref	0.65(0.47–0.88)	0.40(0.23–0.66)
**PAI**				
Low PAI	308/4,892	Ref	0.52(0.37–0.73)	0.57(0.33–0.93)
High PAI	104/5,103	Ref	0.86(0.55–1.33)	0.14(0.04–0.36)
**Smoking**				
Yes	79/2,232	Ref	0.62(0.34–1.09)	0.17(0.39–0.50)
No	333/7,763	Ref	0.64(0.47–0.86)	0.44(0.26–0.70)
**Drinking**				
Yes	72/2,808	Ref	0.79(0.44–1.38)	0.18(0.04–0.52)
No	340/7,187	Ref	0.59(0.44–0.80)	0.44(0.26–0.70)
**Hypertension**				
Yes	358/5,827	Ref	0.56(0.42–0.75)	0.36(0.22–0.57)
No	54/4,168	Ref	1.52(0.74–3.09)	0.51(0.11–1.64)
**Diabetes**				
Yes	122/1,673	Ref	0.57(0.36–0.90)	0.18(0.06–0.43)
No	290/8,322	Ref	0.69(0.49–0.95)	0.50(0.29–0.82)

^a^ Entire represent one category increase of HGS

^b^ Adjusted for age, sex, BMI, smoking, drinking, physical activity index (PAI), history of chronic diseases, HDL, LDL, TG

## Discussion

In this study, we identified that individuals with higher HGS tended to have a lower prevalence of CKD and that HGS was significantly related to the decreased prevalence of CKD. To our knowledge, very few epidemiologic studies have quantified the link between HGS and CKD in community-dwelling Chinese persons.

HGS has traditionally been viewed as a strong predictor of physical performance and is greatly influenced by age and sex [22, 23]. We first produced normative data for HGS across the whole age range (ages 18–95) stratified by males and females. Our study shows that at 20–35 years old, males began to gain strength more rapidly to a higher peak median of 44 kg, compared with the peak female median grip of 23 kg, and these values were, on average, lower than those in the Great Britain study [24], while consistent with those in a Korean cohort [25], suggesting a variation among countries. Furthermore, we found that HGS began to decrease with age from age 50 through centile curves, indicating the threshold age of HGS.

The association between HGS and poor outcomes is well established for patients with CKD, both during and out of the dialysis session [26–30], indicating that implementation of physical activity was warranted and useful both during and out of the dialysis session [31, 32]. In the present study, we identified that HGS was prevalently lower in the old population aged 50 and above, particularly in those affected by CKD. Thus, accurate identification of older adults with lower HGS is important. From our cross-sectional study of males aged ≥ 50 years, the odds of CKD were nearly 2-fold higher in moderate HGS than in high HGS, independent of age, BMI, smoking, drinking, PAI, history of chronic diseases and so on. The strength of this association was similar for women aged ≥ 50 years. Considering these results together, it was speculated that strengthening physical activity exercise and improving grip strength levels may decrease the prevalence of CKD.

In addition, our study has strengths as shown below. First, a large sample size with more than ten thousand participants in a relatively wide range of ages was included in the analyses to increase the statistical power. HGS [33] decreases with age and differs among males and females [34]. Thus, we used GAMLSS to present the age-related, sex-specific standards for HGS and considered categories of HGS through sex-specific tertiles.

One important limitation of our study is that the cross-sectional data limits the ability to draw conclusions about causality. Renal failure itself is a proinflammatory state that contributes to decreased muscle strength [35, 36], while Koji et al. concluded that a decline in indices of physical function had significant associations with eGFR [37]. Therefore, it is unknown whether the decline in handgrip strength worsens kidney function or vice versa. Second, most of the participants were from rural populations, and even strong HGS might be linked to relatively high levels of muscular work with increased exposure to smoking and drinking as well as high levels of lipids. In addition, we examined the association between HGS and CKD, controlling for demographic characteristics such as age and sex, but failed to account for important variables such as marital status, education level, household income and nutritional status. Finally, because of the lack of information on albuminuria, we may be underestimating the prevalence of CKD. Thus, this study may oversimplify the relationship between HGS and CKD.

## Conclusions

This study demonstrated that handgrip strength decreased as kidney function declined, which can be used as a reliable and inexpensive tool in clinical practice to assess the prevalence of CKD. Furthermore, a prospective cohort study is necessary to elucidate the exact changes in handgrip strength associated with the progression of renal dysfunction.

## Supplementary Information

**Additional file 1:**

## Data Availability

The datasets used and analyzed during the current study are available from the corresponding author on reasonable request.

## Abbreviations

CKD: Chronic Kidney Disease
HGS: Handgrip Strength
GAMLSS: General Additive Model for Location Scale and Shape
BP: Blood Pressure
PAI: Physical Activity Index
TG: Triglycerides
HDL-C: High-Density Lipoprotein Cholesterol
LDL-C: Low-Density Lipoprotein Cholesterol
OR: Odds Ratio
CI: Confidence Interval
eGFR: Estimated Glomerular Filtration Rate
BMI: Body Mass Index
Scc: Serum Cystatin C
Scr: Serum Creatinine
TC: Total Cholesterol
