# Supplementary material for: Chronic kidney disease: prevalence and association with handgrip strength in a cross-sectional study
Source: BMC Nephrol. 2021 Jul 2;22:246. doi: 10.1186/s12882-021-02452-5 (PMC8252238; doi:10.1186/s12882-021-02452-5)
Supplement: Supplementary file 1 — Additional file 1: [file 12882_2021_2452_MOESM1_ESM.doc]

**Table S1. Age-related distribution of handgrip strength stratified by sex.**

| **Age (years)** | **Man (kg)** | | | | |  | **Woman(kg)** | | | | |
| --- | --- | --- | --- | --- | --- | --- | --- | --- | --- | --- | --- |
|  | **10th** | **25th** | **50th** | **75th** | **90th** |  | **10th** | **25th** | **50th** | **75th** | **90th** |
| **20** | 31.68 | 38.01 | 44.26 | 50.2 | 55.63 |  | 18.82 | 22.53 | 26.28 | 29.9 | 33.26 |
| **25** | 31.6 | 37.91 | 44.15 | 50.07 | 55.48 |  | 18.93 | 22.66 | 26.44 | 30.08 | 33.46 |
| **30** | 31.48 | 37.76 | 43.97 | 49.87 | 55.26 |  | 19.03 | 22.78 | 26.57 | 30.24 | 33.63 |
| **35** | 31.21 | 37.44 | 43.6 | 49.45 | 54.8 |  | 19.07 | 22.82 | 26.62 | 30.3 | 33.69 |
| **40** | 30.68 | 36.81 | 42.86 | 48.61 | 53.86 |  | 19 | 22.74 | 26.52 | 30.18 | 33.57 |
| **45** | 29.82 | 35.77 | 41.66 | 47.24 | 52.35 |  | 18.76 | 22.46 | 26.2 | 29.81 | 33.16 |
| **50** | 28.63 | 34.34 | 39.99 | 45.36 | 50.26 |  | 18.31 | 21.92 | 25.57 | 29.09 | 32.36 |
| **55** | 27.21 | 32.64 | 38.01 | 43.11 | 47.77 |  | 17.64 | 21.12 | 24.63 | 28.03 | 31.18 |
| **60** | 25.76 | 30.91 | 35.99 | 40.82 | 45.23 |  | 16.83 | 20.15 | 23.5 | 26.75 | 29.75 |
| **65** | 24.28 | 29.12 | 33.91 | 38.46 | 42.62 |  | 15.88 | 19.01 | 22.17 | 25.23 | 28.07 |
| **70** | 22.57 | 27.08 | 31.53 | 35.76 | 39.63 |  | 14.79 | 17.7 | 20.65 | 23.5 | 26.13 |
| **75** | 20.78 | 24.93 | 29.03 | 32.93 | 36.49 |  | 13.55 | 16.22 | 18.91 | 21.53 | 23.94 |
| **80** | 19.07 | 22.88 | 26.64 | 30.21 | 33.48 |  | 12.16 | 14.56 | 16.98 | 19.32 | 21.49 |
| **85** | 17.28 | 20.72 | 24.13 | 27.37 | 30.33 |  | 10.74 | 12.85 | 14.99 | 17.06 | 18.98 |
| **90** | 15.4 | 18.47 | 21.51 | 24.39 | 27.03 |  | 9.3 | 11.13 | 12.99 | 14.78 | 16.44 |

**Table S2. Low Handgrip Strength Prevalence According to CKD**

| **Characteristics** | **Sample** | **Handgrip strength** | | |
| --- | --- | --- | --- | --- |
|  | **(Case/Control)** | **High (n=3483) ^b^** | **Moderate(n=3484) ^b^** | **Low (n=3440) ^b^** |
| **Entire ^a^** | 412/9995 | Ref | 1.71(1.08-2.79) | 2.68(1.74-4.30) |
| **Gender** |  |  |  |  |
| Men | 189/3895 | Ref | 1.96(0.99-4.25) | 3.23(1.67-6.90) |
| Women | 223/6100 | Ref | 1.53(0.84-2.94) | 2.29(1.30-4.30) |
| **Age (≥50)** |  | Ref | 1.95(1.22-3.23) | 5.18(3.39-8.32) |
| Men | 188/3231 | Ref | 2.17(1.07-4.88) | 6.05(3.17-13.08) |
| Women | 221/4750 | Ref | 1.76 (0.95-3.45) | 4.51(2.62-8.48) |
| **BMI (≥28kg/m^2^)** |  |  |  |  |
| Yes | 86/1760 | Ref | 1.98(0.85-5.17) | 3.39(1.52-8.68) |
| No | 326/8235 | Ref | 1.63(0.96-2.92) | 2.51(1.51-4.41) |
| **PAI** |  |  |  |  |
| Low PAI | 308/4892 | Ref | 0.92(0.54-1.64) | 1.77(1.08-3.03) |
| High PAI | 104/5103 | Ref | 6.04(2.39-20.34) | 7.03(2.79-23.66) |
| **Smoking** |  |  |  |  |
| Yes | 79/2232 | Ref | 3.67(1.20-15.96) | 5.91(1.98-25.48) |
| No | 333/7763 | Ref | 1.45(0.88-2.47) | 2.28(1.43-3.80) |
| **Drinking** |  |  |  |  |
| Yes | 72/2808 | Ref | 4.47(1.49-19.28) | 5.66(1.91-24.33) |
| No | 340/7187 | Ref | 1.36(0.82-2.32) | 2.28(1.43-3.81) |
| **Hypertension** |  |  |  |  |
| Yes | 358/5827 | Ref | 1.57(0.96-2.66) | 2.77(1.75-4.61) |
| No | 54/4168 | Ref | 2.95(0.94-1.31) | 1.95(0.61-8.70) |
| **Diabetes** |  |  |  |  |
| Yes | 122/1673 | Ref | 3.16(1.29-9.48) | 5.51(2.35-16.16) |
| No | 290/8322 | Ref | 1.38(0.82-2.42) | 2.00(1.22-3.47) |

^a^ Entire represent one category increase of HGS.

^b^ Adjusted for age, sex, BMI, smoking, drinking, physical activity index (PAI), history of chronic diseases, HDL, LDL, TG.

**Fig.S1.The distributions of handgrip strength (A1-A3) in different chronic kidney disease stages.**

**
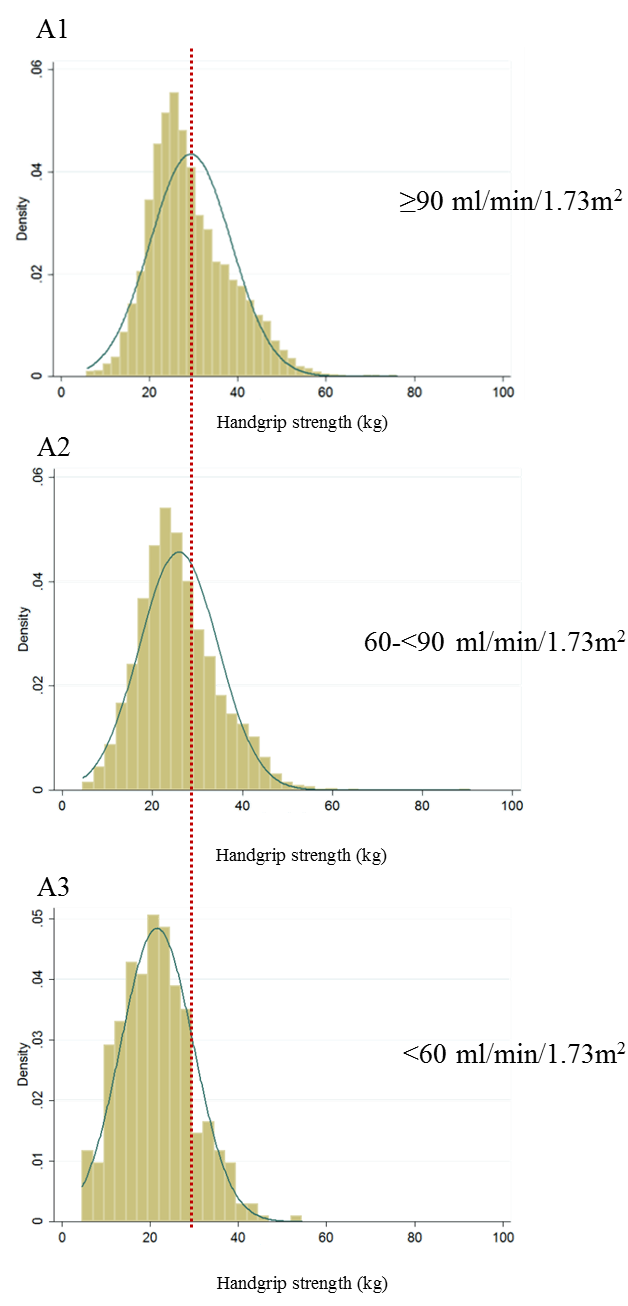
**
